# Double Knockdown of PHD1 and Keap1 Attenuated Hypoxia-Induced Injuries in Hepatocytes

**DOI:** 10.3389/fphys.2017.00291

**Published:** 2017-05-10

**Authors:** Jing Liu, Yiping Li, Lei Liu, Zhi Wang, Chuanbing Shi, Zhengyuan Cheng, Xiaoyi Zhang, Fengan Ding, Ping Sheng Chen

**Affiliations:** Department of Pathology and Pathophysiology, School of Medicine, Southeast UniversityNanjing, China

**Keywords:** liver fibrosis, hepatocytes, PHD1, Keap1, hypoxia, oxidative stress

## Abstract

**Background and Aims:** Hypoxia and oxidative stress contribute toward liver fibrosis. In this experiment, we used small hairpin RNA (shRNA) to interfere with the intracellular oxygen sensor—prolyl hydroxylase 1 (PHD1) and the intracellular oxidative stress sensor—kelch-like ECH associated protein 1 (Keap1) in the hypoxic hepatocytes in order to investigate the function of PHD1and Keap1.

**Methods:** We first established the CCl_4_-induced liver fibrosis model, subsequently, the levels of the PHD1, hypoxia-inducible factor-1α (HIF-1α), hypoxia-inducible factor-2α (HIF-2α), Keap1, and nuclear factor-erythroid 2 p45-related factor 2 (Nrf2) were detected in liver tissues. Simultaneously, AML12 cells co-transfected with PHD1 and Keap1shRNAs were constructed *in vitro*, then the intracellular oxidative stress, the proportion of cells undergoing apoptosis, and cell viability were measured. The expression of pro-fibrogenic molecules were analyzed via quantitative real-time polymerase chain reaction (qRT-PCR) and western blot. The level of alpha-1 type I collagen (COL1A1) was determined using an enzyme-linked immunosorbent assay (ELISA). Finally, serum-free “conditioned medium” (CM) from the supernatant of hypoxic AML12 hepatocytes was used to culture rat hepatic stellate cells (HSC-T6), and the levels of fibrosis-related molecules, apoptosis, and cell proliferation were determined.

**Results:** The marker of hypoxia—HIF-1α and HIF-2α in the livers with fibrosis were upregulated, however, the increase in PHD1 expression was not statistically significant in comparison to the control group. Sign of oxidative stress—Keap1 was increased, while the expression of Nrf2, one of the Keap1 main downstream molecules, was reduced in the hepatocytes. And *in vitro*, the double-knockdown of PHD1 and Keap1 in AML12 hepatocytes presented with decreased hypoxia-induced oxidative stress and apoptosis, furthermore, these hypoxic AML12 cells showed the increased cell viability and the doweregulated expression of pro-fibrogenic molecules. In addition, HSC-T6 cells cultured in the hypoxic double-knockdown CM demonstrated the downregulation of fibrosis-related molecules, diminished cell proliferation, and enhanced apoptosis.

**Conclusions:** Our study demonstrated that double-knockdown of PHD1 and Keap1 attenuated hypoxia and oxidative stress induced injury in the hepatocytes, and subsequently inhibited HSC activation, which offers a novel therapeutic strategy in the prophylaxis and treatment of liver fibrosis.

## Introduction

Liver fibrosis is a pathological process caused by numerous chronic liver damages—such as viral hepatitis, alchoholic hepatitis, and drug induced hepatotoxicity (Begriche et al., 2011). As a highly active metabolic organ, the liver is particularly prone to hypoxic environments and the damages caused by it (Nakanishi et al., 1995). A convincing body of evidence suggest that hypoxia plays an important role in the pathogenesis of liver fibrosis (Cannito et al., 2014).

Cellular hypoxia leads to activation of hypoxia-inducible factors (HIF), which is crucial for the survival of an asphyxiated/ischemic hepatocyte. In normoxia, HIF-1α and HIF-2α are first hydroxylated by prolyl hydroxylase (PHD) and then degraded by proteasomes (Kamura et al., 2000). Whereas during hypoxic condition, HIF-1α and HIF-2α accumulate and get translocated to the nuclei, thereby activating the genes responsible for limiting hypoxia-induced injury and cell death. Recently, Nimker et al. reported that the pharmaceutical inhibition of PHDs with ethyl 3, 4-dihydroxy benzoate (EDHB) protects myoblasts against hypoxia-induced oxidative damage by upregulating HIFs (Nimker et al., 2015). However, non-specific inhibition of PHDs also induces the activation of HIFs but at the cost of adverse effects such as steatosis (Minamishima et al., 2009; Rankin et al., 2009). PHD has three isoforms—PHD1, PHD2, and PHD3, each of these has numerous functions. Selective loss of PHD1, but not those of PHD2 or PHD3 could induce hypoxia tolerance in the skeletal muscle and liver cells via reprogramming of basal oxygen metabolism without inducing angiogenesis and erythrocytosis (Aragones et al., 2008; Schneider et al., 2010). Therefore, the specific inhibition of PHD1 could serve as a potential therapeutic strategy against hypoxia during liver fibrosis.

In recent years, many studies have demonstrated that oxidative stress also contributes to the pathogenesis of liver fibrosis (Ghatak et al., 2011; Mormone et al., 2012; Yang et al., 2013). The nuclear factor-erythroid 2-related factor 2 (Nrf2) has been suggested to be involved in this process. Under normal condition, Nrf2 exists in the cytoplasm where it binds to kelch-like ECH associated protein 1 (Keap1), thereby hastening the Nrf2 ubiquitination and degradation (Kang et al., 2004). During oxidative stress, Nrf2 evades Keap1 and translocates to the nucleus, where it activates antioxidant genes. Chen et al. has also demonstrated that glycyrrhetinic acid could ameliorate chronic liver fibrosis via upregulation of Nrf2 (Chen et al., 2013). Additonally, Tanaka et al. also reported that Nrf2-null mice had an increased susceptibility to liver injury (Tanaka et al., 2012). Keap1 serves to negatively regulate Nrf2 (Miyata et al., 2011), for example, Keap1-knockdown mice are less vulnerable to oxidative liver injuries during obstructive cholestasis mediated by enhanced expression of Nrf2 (Okada et al., 2009). Thus, Keap1 may be an efficient therapeutic target for relieving oxidative stress injury during liver fibrosis.

It has been reported that many cell types are involved in the pathogenesis of liver fibrosis, activated hepatic stellate cells (HSCs) play a central role in liver fibrogenesis. It is worth noting that injuried hepatocytes are considered to be the primary activator of HSCs (Nieto et al., 2002), and hepatocytes are the major parenchymal cells that account for more than 70% of all liver cells (Bogdanos et al., 2013). Furthermore, hepatocytes are highly susceptible to hypoxic injury and the drugs induced toxicity, therefore we chose hepatocytes as a therapeutic target in an attempt to ameliorate liver fibrosis. Hypoxic hepatocytes are known to generate excessive amount of reactive oxygen species (ROS) which exert oxidative stress on the liver. Moreover, such oxidative stress can further exacerbate the hypoxia in the hepatocytes, and the subsequent re-oxygenation following hypoxia leads to additional oxidative stress (Miyata et al., 2011). Therefore, the ideal therapy for liver fibrosis could be the alleviation of hypoxia along with the oxidative stress, however, no such related studies have been done so far.

RNA interference (RNAi) is a method that uses a small complementary double-stranded RNA (dsRNA) molecule to silence a target gene (Inoue et al., 2006). Considering the short half-life and high cost of current small interfering RNA (siRNA) vectors, they are not suitable for many *in vivo* studies. In contrast, short hairpin RNA (shRNA) is widely used due to its enhanced stability and transfer efficiency. Accordingly, for the present study we used shRNAs to simultaneously knockdown the expressions of PHD1 and Keap1 in the hepatocytes with the aim of exploring therapeutic target for liver fibrosis.

## Materials and methods

### Animals and treatment

Six-week-old male Sprague-Dawley rats (250 ± 30 g) were purchased from the Animal Center of Yangzhou University (Yangzhou, China). All animals were housed in the animal experimental center of Southeast University under constant temperature (22°C) and humidity (55 ± 5%) in a controlled room with a 12–12 h light-dark cycle, where the diet and water were available *ad libitum*. The rats were acclimatized under these conditions for at least 1 week prior to experiments. All animals were randomly divided into two groups: a treatment group (*n* = 10) which received 1.5 ml/kg body weight carbon tetrachloride (CCl_4_; 40% CCl_4_ in olive oil) via intraperitoneal injection twice a week for eight weeks and a normal control group (*n* = 10) which received a saline injection in parallel. This study was carried out in accordance with the recommendations of the European Council Directive of the 24th November 1986 (86/609/EEC). All procedures were approved by the Animal Research Ethics Committee at the Medical School of the Southeast University (Nanjing, China).

### Histology and immunohistochemistry

Excised livers were fixed in 10% neutral-buffered formalin, embedded in paraffin, and cut into sections of 5 μm thickness. Slides were then deparaffinized with dimethylbenzene, dehydrated with graded ethanol, and stained with hematoxylin and eosin (H&E) and Masson dyes to evaluate the degree of liver fibrosis.

For immunohistochemical probing, liver slides were initially boiled in a pressure cooker containing a citric acid buffer (pH 6.0) to retrieve antigens. Then the slides were blocked with 5% BSA and incubated with primary antibodies (Table 1) overnight at 4°C, and with a goat anti-rabbit biotinylated secondary antibody for 20 min at 37°C. Immunolabels were detected with 3, 3-Diaminobenzidine (DAB), after which the nuclei were counterstained with hematoxylin. Slides were inspected under a fluorescence microscope at 200× magnification. For quantification analysis, the percentage of positive area for immunohistochemistry was determined using ImageJ software.

**Table 1:** **The antibody for immunohistochemistry and western blot**.

**Antibody**	**Manufacturer**	**Cat. No**.
HIF-1α	Abcam	ab179483
HIF-2α	Abcam	ab179825
PHD1	Abcam	ab108980
Nrf2	Abcam	ab137550
Keap1	Proteintech	10503-2-AP
α-SMA	Proteintech	14395-1-AP
COL1A1	Bioworld	BS60771-25
TGF-β1	Bioworld	BS1361
VEGF-A	Proteintech	19003-1-AP
IGF-1	Bioworld	BS2909
GAPDH	Bioworld	AP0063

### Cell cultures and hypoxia treatment

AML12 (alpha mouse liver 12 cells) and HSC-T6 (rat hepatic stellate cells) were purchased from the Shanghai Institute of Biochemistry and Cell Biology. Cells were cultured in DMEM-F12 (Hyclone, USA) supplemented with 10% heat-inactivated fetal bovine serum (Bioind, Israel), and were maintained in a humidified atmosphere with 5% CO_2_ at 37°C. For hypoxic exposure, AML12 cells were incubated in a tri-gas incubator (Thermo, America) of 1% O_2_, 5% CO_2_, and 94% N_2_. Serum-free “conditioned medium” (CM) was obtained from the supernatant of AML12 cells to eliminate the influence of serum cytokines. HSC-T6 cells were cultured in CM and the cells cultured in DMEM were used as control.

### RNA isolation and quantitative real-time polymerase chain reaction

Total RNA was extracted from cells with Trizol reagent (TaKaRa, Japan). Then, 1 μg RNA was added to a 20 μl reaction volume for cDNA reverse transcription using the Prime Script™ RT reagent Kit with gDNA Eraser (TaKaRa, Japan), and quantitative real-time polymerase chain reaction (qRT-PCR) was performed using the BR® Premix Ex Taq™ (TaKaRa, Japan) in a Step One Plus real-time PCR system (Applied Biosystems, USA). Gene expression was quantified according to the 2^−ΔΔCt^ method. All PCR primers are listed in Table 2.

**Table 2:** **Primer sequences for quantitative PCR**.

**Specie**	**Genes**	**Forward sequence (5′–3′)**	**Reverse sequence (5′–3′)**
Mouse	Keap1	TGCCCCTGTGGTCAAAGTG	AGTCCTTGGAGTCTAGCCGAG
	Nrf2	TCTTGGAGTAAGTCGAGAAGTGT	GTTGAAACTGAGCGAAAAAGGC
	PHD1	AGTCCTTGGAGTCTAGCCGAG	GGTTCGGTTACCGTCCTGC
	HIF-1α	GATGACGGCGACATGGTTTAC	CTCACTGGGCCATTTCTGTGT
	HIF-2α	TCCTTCGGACACATAAGCTCC	GACAGAAAGATCATGTCACCGT
	COL1A1	GCTCCTCTTAGGGGCCACT	CCACGTCTCACCATTGGGG
	VEGF-A	GCACATAGAGAGAATGAGCTTCC	CTCCGCTCTGAACAAGGCT
	TGF-β1	CTCCCGTGGCTTCTAGTGC	GCCTTAGTTTGGACAGGATCTG
	IGF-1	CACCCTGTGACCTCAGTCAA	CAAGGGTTCTGATGTTGCAC
	GAPDH	TGGCCTTCCGTGTTCCTAC	GAGTTGCTGTTGAAGTCGCA
Rat	COL1A1	GTACATCAGCCCAAACCCCA	CAGGATCGGAACCTTCGCTT
	α-SMA	GGAGATGGCGTGACTCACAA	CGCTCAGCAGTAGTCACGAA
	TGF-β1	AGGGCTACCATGCCAACTTC	CCACGTAGTAGACGATGGGC
	VEGF-A	CGGGCCTCTGAAACCATGAA	GCTTTCTGCTCCCCTTCTGT
	IGF-1	CAGTTCGTGTGTGGACCAAG	TCAGCGGAGCACAGTACATC
	GAPDH	GAAGGGCTCATGACCACAGT	GGATGCAGGGATGATGTTCT

### Protein extraction and western blot

Cells were lysed with RIPA buffer (Beyotime, China) containing a Protease Inhibitor Cocktail (Roche, Germany) and then centrifuged at 12,000 × g for 15 min at 4°C. Equal amounts of protein were separated via gel electrophoresis and transferred to a PVDF membrane (Millipore Corp, USA). Membranes were blocked with 5% skimmed milk for 1 h at 37°C and incubated with primary antibodies (Table 1) overnight at 4°C and then with corresponding secondary antibodies for 1 h at 37°C. The blotting signal was visualized using enhanced chemiluminescence reagent (HaiGene, China). Final protein levels were normalized to that of GAPDH.

### Malondialdehyde (MDA), reduced glutathione (GSH) levels, and lactate dehydrogenase (LDH) activity assay

MDA and GSH levels were detected in total cell lysates via commercial assay kits (Jian Cheng Bioengineering Institute, China). All levels are expressed as μmol/g protein. For detection of LDH activity, supernatants were collected and analyzed with the LDH assay kit (Jian Cheng Bioengineering Institute, China). LDH activity is expressed as U/L.

### Enzyme-linked immunosorbent assay for collagen secretion

The level of alpha-1 type I collagen (COL1A1) in the supernatants of AML12 cells was determined using a commercial enzyme-linked immunosorbent assay (ELISA) kit (Hengyuan Biology, China) according to the manufacturer's protocol. The optical density was measured at a wavelength of 450 nm.

### Cell viability assay

Cellular viability was measured using the cell counting kit-8 (CCK-8) assay (Dojindo, Japan) following the manufacturer's protocol. Absorbance (OD value) was measured at 450 nm using an enzyme-linked immunosorbent assay reader (Thermo Fisher Scientific).

### Apoptosis assay

Cells undergoing apoptosis were stained with the Annexin-V/PI stain (Ebscience, America). For cells that were transfected with plasmids with green and red fluorescence, PI was replaced by DAPI to label apoptosis. A Flow cytometer (Becton Dickinson, USA) was used to quantify apoptotic cells.

### Statistical analysis

Results are expressed as mean ± SEM. One-way ANOVA and the Student's *t*-test were used to evaluate any variations in outcomes between different groups. A *p*-value < 0.05 was considered as statistically significant.

## Results

### Hypoxia and oxidative stress in the rats with liver fibrosis

**Figure 1:**
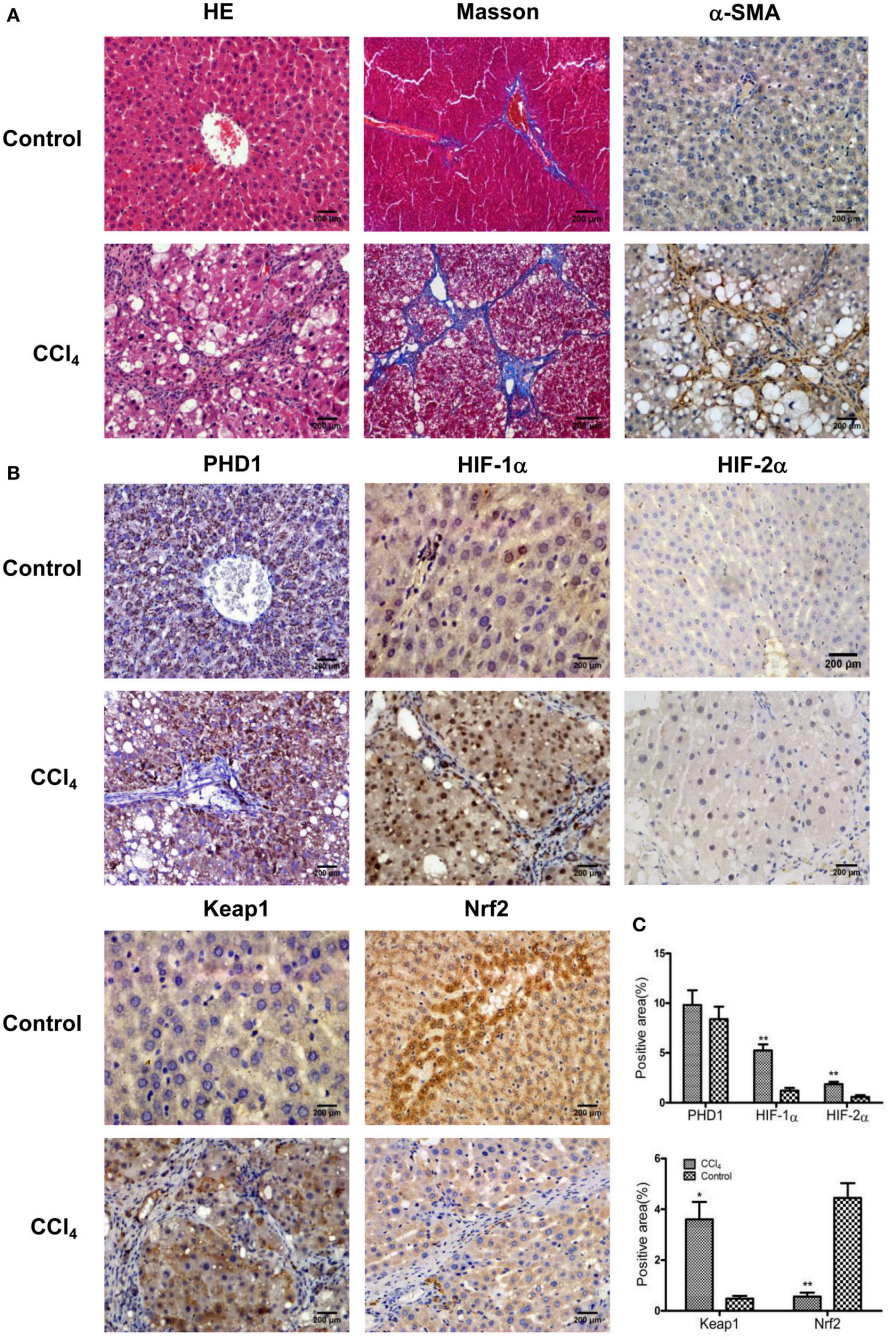
**CCl_4_ upregulated the expression of Keap1, HIF-1α, and HIF-2α but decreased Nrf2 in the liver tissues**. Hepatic morphology as evaluated by H&E and Masson staining **(A)**. The expression of α-SMA **(A)**, PHD1, HIF-1α, HIF-2α, Keap1, and Nrf2 **(B)** were measured by immunohistochemical staining. The percentage of positive area were quantified **(C)**. The scale bar represents 200 μm. ^*^p < 0.05, ^**^p < 0.01 vs. control.

To understand the microenvironment in the fibrotic liver tissues, we established CCl_4_-induced liver fibrosis rat model. H&E staining showed that CCl_4_ exposure resulted in severe damage of the liver cells, including hepatocytes necrosis, inflammatory cells infiltration, steatosis, and fibrosis connective tissues proliferation. However, the control group presented normal histological morphology (Figure 1A). Masson's trichrome staining imparts a blue color to collagen against the red background of other structures. According to the microscopic examination, CCl_4_ treatment resulted in an increased collagen deposition around the portal area and formation of septal fibrosis (Figure 1A). Furthermore, immunohistochemical staining demonstrated an increased expression of α-smooth muscle actin (α-SMA, a marker of HSC activation, which plays a critical role in liver fibrogenesis) in fibrous septae (Figure 1A). These results indicated that the CCl_4_-induced liver fibrosis was conducted successfully. Sitmultaneously, we found the upregulation of HIF-1α and HIF-2α (markers of hypoxia), and Keap1 (an intracellular oxidative stress sensor) in hepatocytes in response to CCl_4_ treatment (Figures 1B,C). However, Nrf2, one of the main downstream molecules of Keap1, was downregulated, while PHD1 showed no obvious change (Figures 1B,C).

### Effects of PHD1shRNA on HIF levels in the hypoxic AML12 cells

**Figure 2:**
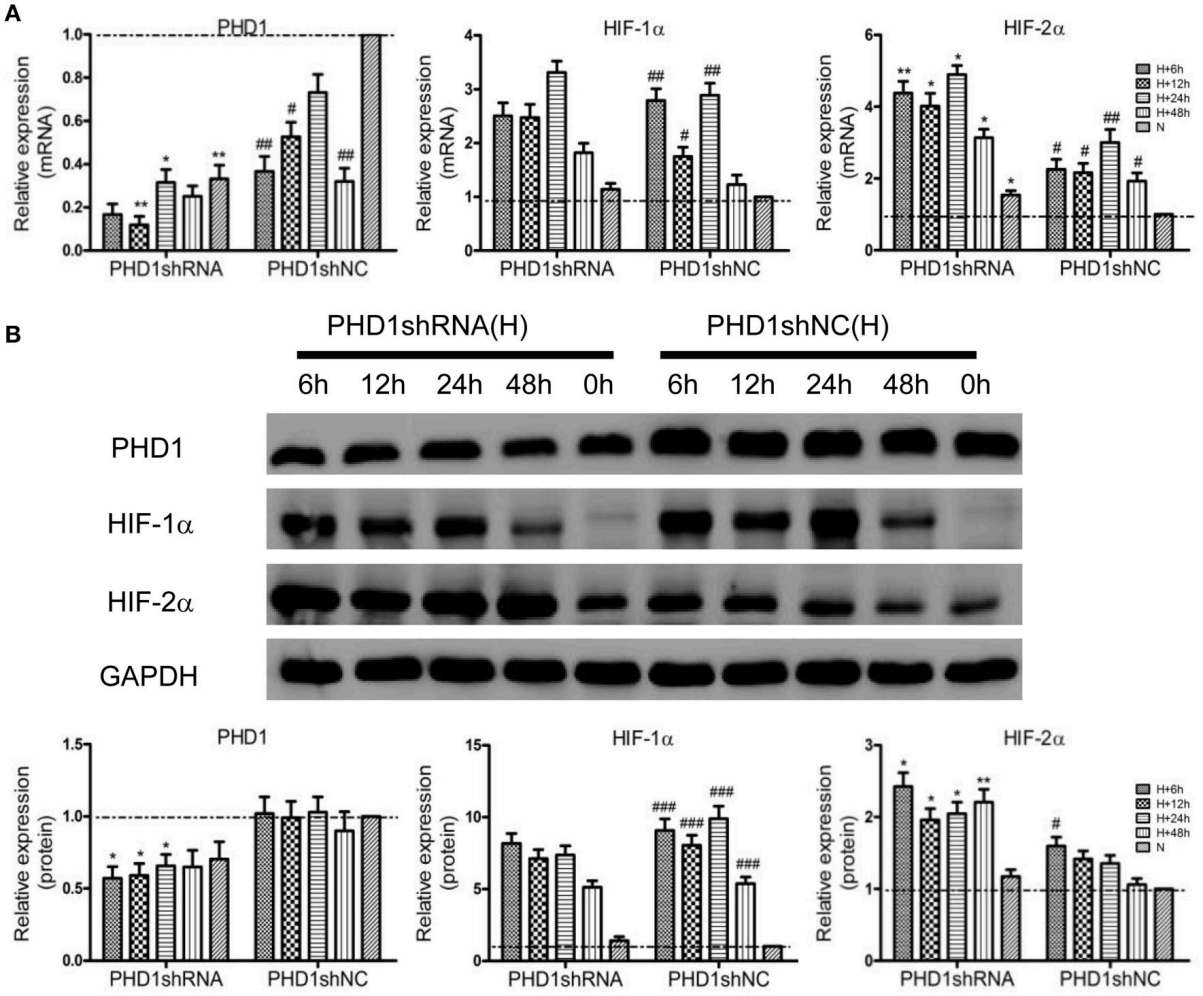
**PHD1shRNA upregulated the expression of HIFs in hypoxic AML12 cells at various time interval between 0–48 h**. **(A)** mRNAs and **(B)** proteins levels of PHD1, HIF-1α and HIF-2α were measured by qRT-PCR and western blot, and GAPDH was used as the internal standard, *n* = 3. ^*^p < 0.05, ^**^p < 0.01 vs. shNC at equivalent time interval, ^#^*p* < 0.05, ^##^*p* < 0.01, ^###^*p* < 0.001 vs. shNC under normoxia. N, normoxia; H, hypoxia.

To clarify the function of PHD1 during hypoxia-induced liver injuries, PHD1shRNA was applied to transfect the AML12 cells, then the cells were exposed to hypoxia for 0, 6, 12, 24, and 48 h, the expression of PHD1, HIF-1α and HIF-2α mRNAs and proteins were measured. As shown in Figure 2A, PHD1mRNA expression was reduced in hypoxic shNC (negative control) cells compared to that in normoxic shNC cells. Introduction of PHD1shRNA inhibited 60–80% of PHD1 mRNA expression, achieving optimum gene silencing at 12 h of hypoxia. However, PHD1 protein level was invariably reduced by ~50% regardless of the duration of hypoxia (Figure 2B). Under normoxic condition, the expression of HIF-1α and HIF-2α mRNAs (Figure 2A) and proteins (Figure 2B) were in low expression, and upon exposed to hypoxia, these expression were upregulated. Furthermore, PHD1 silencing further increased the expression of HIF-2α but not that of HIF-1α at both the mRNA and protein levels.

### Effects of Keap1shRNA on Nrf2 level in the hypoxic AML12 cells

**Figure 3:**
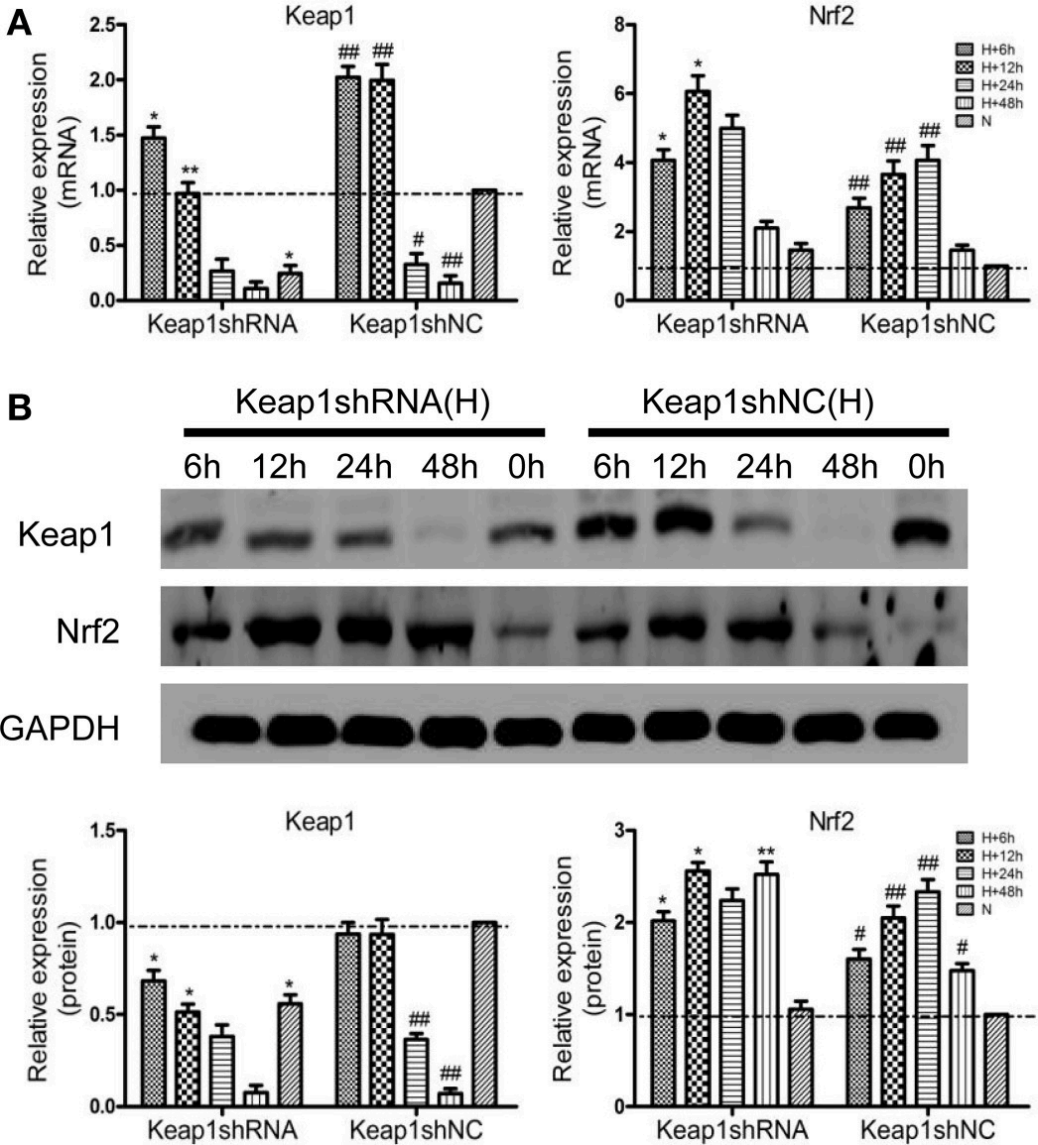
**Keap1shRNA upregulated the expression of Nrf2 in hypoxic AML12 cells at various time interval between 0–48 h**. **(A)** mRNAs and **(B)** proteins levels of Keap1 and Nrf2 were assayed by qRT-PCR and western blot, and GAPDH was used as the internal standard, *n* = 3. ^*^p < 0.05, ^**^p < 0.01 vs. shNC at equivalent time interval, ^#^*p* < 0.05, ^##^*p* < 0.01 vs. shNC under normoxia. N, normoxia; H, hypoxia.

To clarify the effect of Keap1shRNA, the AML12 cells were transfected with Keap1shRNA and were administrated with hypoxia for 0, 6, 12, 24, and 48 h, then the expression of Keap1 and Nrf2 at the mRNA and protein levels was determined. An 84% reduction in Keap1 mRNA (Figure 3A) and 50% reduction in Keap1 protein (Figure 3B) levels were observed at 12 h of hypoxia in the cells silenced with Keap1shRNA. As expected, Nrf2 mRNA and protein levels were increased in the hypoxic shNC cells compared to that in the normoxic shNC cells, and the expression of Nrf2 mRNA and protein were further increased in hypoxic cells treated with Keap1shRNA, which peaked at 12 h of hypoxia (Figures 3A,B).

### Effects of PHD1 and Keap1shRNAs on oxidative stress in the hypoxic AML12 cells

**Figure 4:**
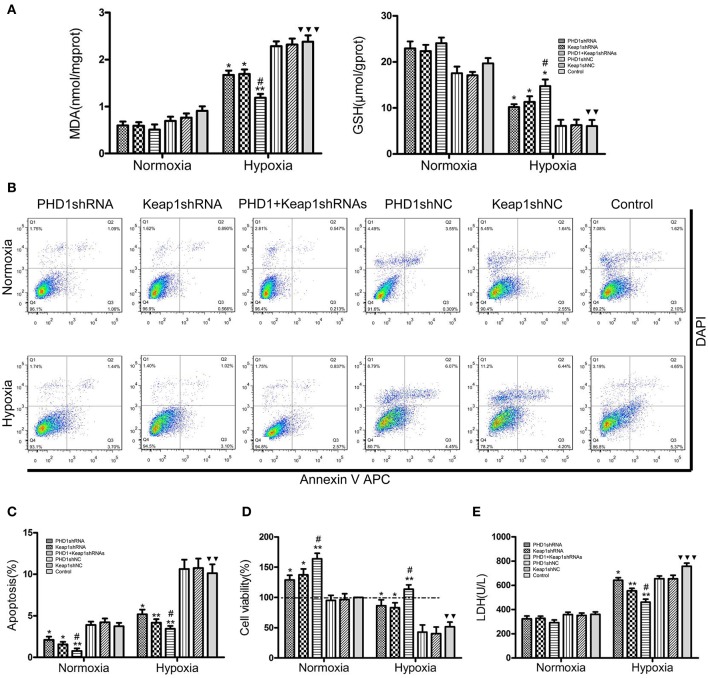
**Co-transfection of PHD1 and Keap1shRNAs inhibited oxidative stress and increased cell viability during hypoxia**. Co-transfected AML12 cells were exposed to hypoxia for 12 h, and **(A)** intracellular MDA and GSH levels, **(B,C)** Annexin V-APC/DAPI double-stained cells undergoing apoptosis, **(D)** cell viability and **(E)** LDH levels were assessed. Data are expressed as mean ± SEM, *n* = 3. ^*^p < 0.05, ^**^p < 0.01 vs. respective normoxic and hypoxic control, ^#^*p* < 0.05 vs. respective single-knockdown normoxic and hypoxic control, ^▾▾^*p* < 0.01, ^▾▾▾^*p* < 0.001 vs. normoxic control.

Since hypoxia led to an increase in oxidative stress, we next studied how molecular markers of oxidative stress—MDA and GSH (important lipid peroxidation and endogenous antioxidant marker, respectively) were regulated. The AML12 hepatocytes were co-transfected with PHD1 and Keap1shRNAs, and then were cultured for 12 h under hypoxia. When cells exposed to hypoxia, a dramatic enhancement of MDA was observed, but the treatment of single knockdown of PHD1 or Keap1 suppressed the increase, furthermore, the double knockdown group was more effective in reducing the hypoxia-induced MDA elevation (Figure 4A). However, no significant differences in the levels of MDA and GSH between gene knockdown cells and control cells during normoxia (Figure 4A), The intracellular GSH concentration presented the inverse correlation with the MDA level (Figure 4A).

### Effects of PHD1 and Keap1shRNAs on AML12 cells viability during hypoxia

To understand the effects of PHD1 and Keap1 molecules on cell biological behavior, the Annexin V-APC/DAPI double staining was used to analyze cell apoptosis. The results were presented as the sum of the percentage of early apoptotic cells and late apoptotic cells. In Figures 4B,C, during hypoxia, the percentage of apoptotic cells in the single PHD1shRNA (6.49 ± 0.50%) or Keap1shRNA (5.86 ± 0.45%) treatment group were notably lower than in the hypoxic control group (10.14 ± 0.79%). Moreover, the apoptotic rate was further reduced in the double-knockdown group (4.63 ± 0.36%). A similar trend among treatment and control groups were also observed during normoxia.

Next, the cell viability was examined using the CCK-8 assay. The data in Figure 4D indicated that hypoxia evidently reduced cell viability, however, co-treatment with PHD1 and Keap1 shRNAs led to an increase in cell viability compared to single shRNA transfection. Cellular membrane integrity was analyzed by detecting LDH release. As shown in Figure 4E, LDH release in different hypoxia groups fluctuated with a similar tendency as MDA levels. During normoxia, we found no significant difference in LDH levels between the gene silencing and normoxic control groups. In hypoxia, PHD1shRNA or Keap1shRNA treatment group led to a decrease in LDH release, furthermore, the LDH level of the co-transfected group was lower than that of the single groups.

### Effects of PHD1 and Keap1shRNAs on the expression of pro-fibrogenic molecules in the hypoxic AML12 cells

**Figure 5:**
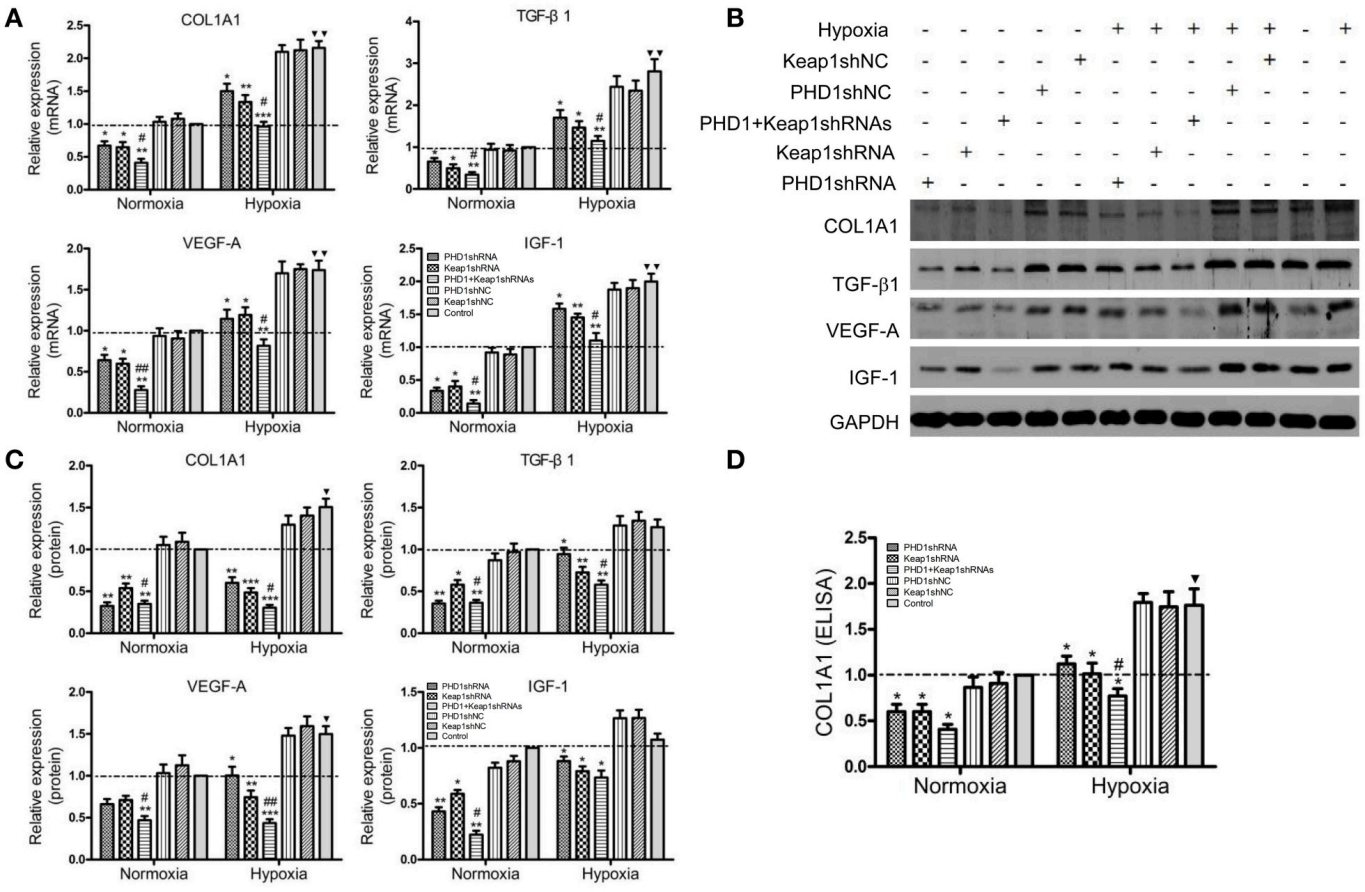
**Co-transfection of PHD1 and Keap1shRNAs inhibited expressions of pro-fibrogenic molecules during hypoxia**. AML12 cells were co-transfected with PHD1 and Keap1 shRNAs, and then exposed to hypoxia for 12 h. **(A)** mRNAs and **(B,C)** proteins levels of COL1A1, TGF-β1, VEGF-A, and IGF-1 were detected by qRT-PCR and western blot. **(D)** COL1A1 level was measured by ELISA. GAPDH was used as the internal standard, *n* = 3. ^*^p < 0.05, ^**^p < 0.01, ^***^p < 0.001 vs. respective normoxic and hypoxic control, ^#^*p* < 0.05, ^##^*p* < 0.01 vs. respective single-knockdown normoxic and hypoxic control, ^▾^*p* < 0.05, ^▾▾^*p* < 0.01 vs. normoxic control.

To clarify the function of PHD1 and Keap1 during fibrogenesis, the expression of some pro-fibrogenic molecules including COL1A1, transforming growth factor-β1 (TGF-β1), vascular endothelial growth factor-A (VEGF-A), and insulin-like growth factor 1 (IGF-1) was evaluated by qRT-PCR and western blot in the hypoxic AML12 cells. Upon exposure to hypoxia, mRNAs (Figure 5A) as well as proteins (Figures 5B,C) levels of COL1A1, TGF-β1, VEGF, and IGF-1 were upregulated, but the changes in TGF-β1 and IGF-1 proteins were not statistically significant.

During hypoxia, single knockdown of PHD1 or Keap1 induced the downregulation of mRNAs (Figure 5A) and proteins (Figures 5B,C) levels of COL1A1, TGF- β1, VEGF-A, and IGF-1 compared to the control. More interestingly, the decrease was further enhanced when PHD1 and Keap1shRNAs were simultaneously introduced. To know whether the release of COL1A1 levels were affected by PHD1 and Keap1shRNAs, an ELISA assay was performed using the AML12 cells culture supernatants. The results showed that the levels of COL1A1 in the supernatants had the same trend of reduction as those in the cell lysates (Figure 5D).

### Effects of PHD1 and Keap1shRNAs-treated hypoxic AML12 cells on the expression of fibrosis-related molecules in HSCs

**Figure 6:**
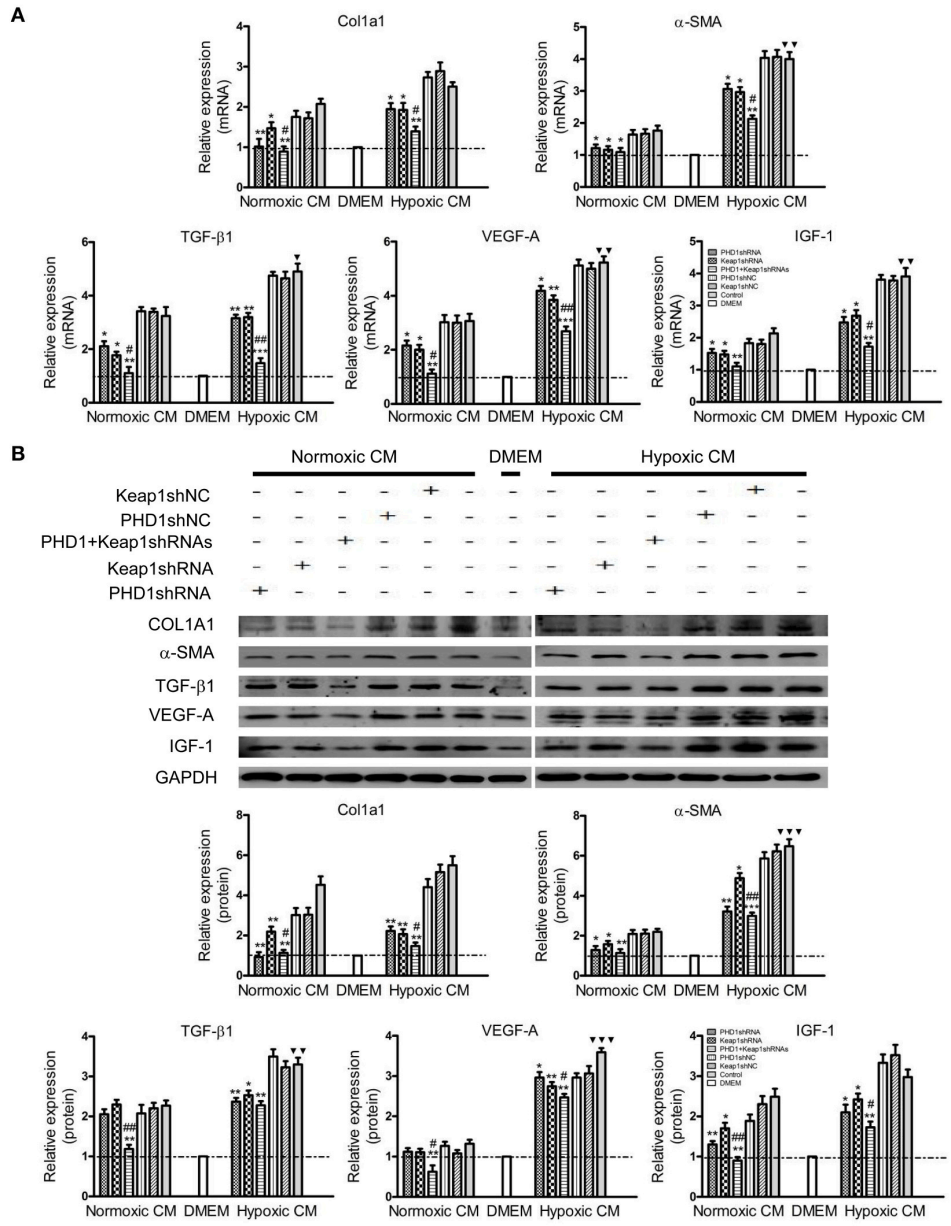
**Effects of PHD1 and Keap1shRNAs-treated hypoxic AML12 cells on the expression of fibrosis-related molecules**. HSC-T6 cells were cultured in CM obtained from the supernatant of hypoxic PHD1 and/or Keap1 knockdown AML12 cells. **(A)** mRNAs and **(B)** proteins levels of COL1A1, α-SMA, TGF-β1, VEGF-A, and IGF-1 were measured via qRT-PCR and western blot. GAPDH was used as the internal standard, *n* = 3. ^*^p < 0.05, ^**^p < 0.01, ^***^p < 0.001 vs. respective normoxic and hypoxic control CM, ^#^*p* < 0.05, ^##^*p* < 0.01 vs. respective normoxic and hypoxic single-knockdown CM, ^▾^*p* < 0.05, ^▾▾^*p* < 0.01, ^▾▾▾^*p* < 0.001 vs. normoxic control CM.

Considering the complex intrahepatic microenvironment, especially the complicated relationship between hepatocytes and HSCs, HSC-T6 cells were cultured in double-knockdown CM to better simulate the *in vivo* local environment, and then the corresponding fibrosis-related molecules were examined at the mRNAs and proteins levels. During hypoxia CM, COL1A1, α-SMA, TGF-β1, VEGF-A, and IGF-1 mRNAs (Figure 6A) and proteins (Figure 6B) in hypoxic single-knockdown CM were lower than those in the hypoxic control CM, and were further decreased in double-knockdown CM. However, no significant difference in these mRNAs and proteins expression was observed between the PHD1 or Keap1shNC CM and the control CM group.

### Effects of PHD1 and Keap1shRNAs-treated hypoxic AML12 cells on apoptosis and proliferation of in HSCs

**Figure 7:**
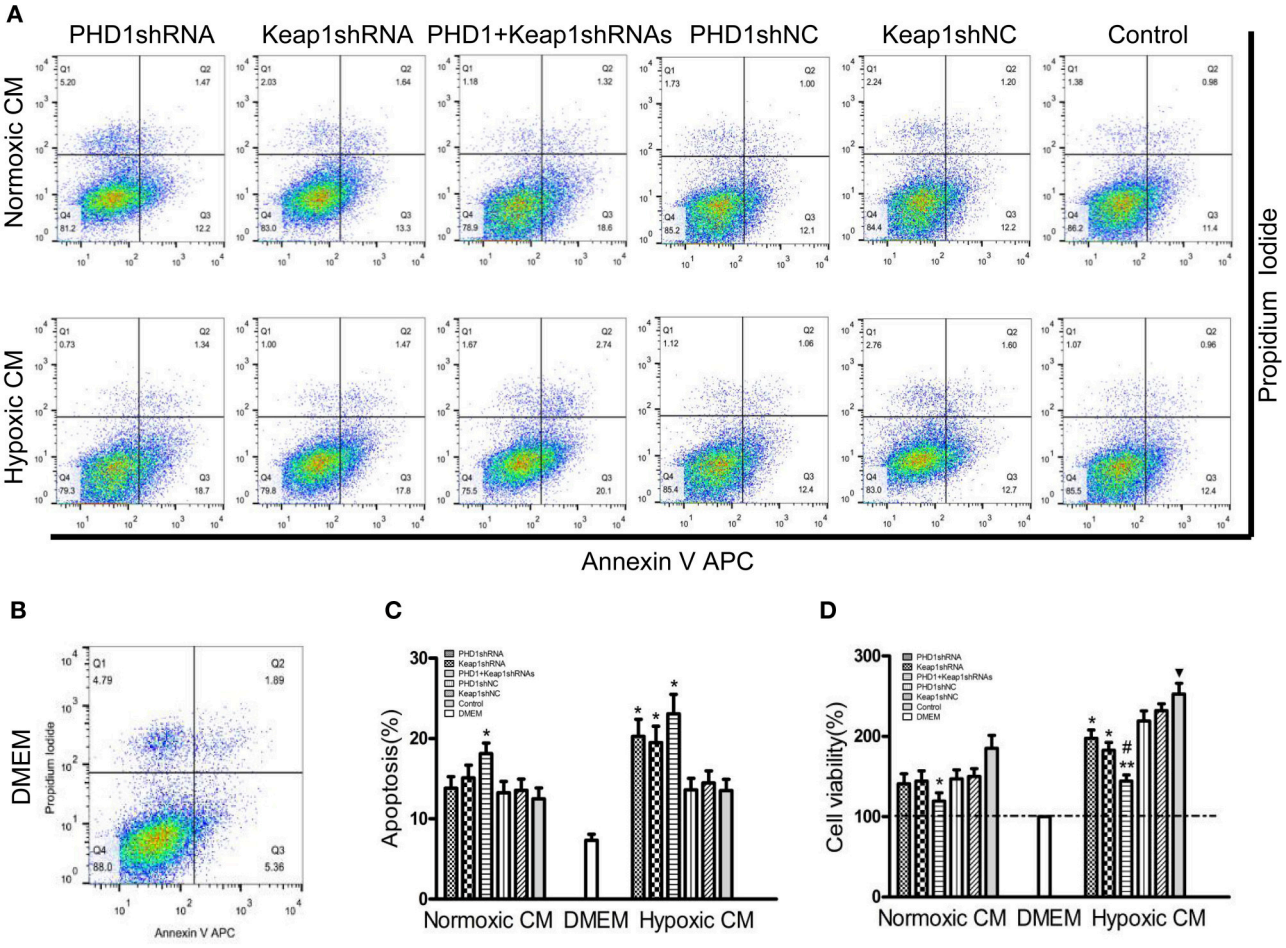
**Effects of PHD1 and Keap1shRNAs-treated hypoxic AML12 cells on the apoptosis and proliferation of HSCs**. HSC-T6 cells were cultured in the CM obtained from the supernatant of hypoxic PHD1 and/or Keap1 AML12 cells. **(A–C)** AnnexinV-APC/PI double-stained cells undergoing apoptosis. **(D)** Cell viability was assessed by CCK-8 assay. Data are expressed as mean ± SEM, *n* = 3. ^*^p < 0.05, ^**^p < 0.01 vs. respective normoxic and hypoxic control CM, ^#^*p* < 0.05 vs. respective normoxic and hypoxic single-knockdown CM, ^▾^*p* < 0.05 vs. normoxic control CM.

Since double-knockdown CM deregulated the expression of fibrosis-related molecules in HSCs, we next detected how the apoptosis of HSCs was regulated using Annexin V-APC/PI double staining. Apoptosis was increased in hypoxic single-knockdown CM, moreover, the apoptotic rate was further enhanced in both normoxic and hypoxic double-knockdown CM (Figures 7A–C). However, there was no significant difference between the knockdown CM groups and control CM group in normoxic CM. In addition, Figure 7D showed that cells cultured in CM enhanced cell viability compared with cells in DMEM, and the effect was enhanced in hypoxic CM. However, the cell proliferation of the HSC-T6 cells was reduced when cultured in single-transfected CM and was further weakened in the co-transfected CM.

## Discussion

In this study, we first successfully established a model of liver fibrosis induced by CCl_4_, which is a suitable model for exploring the underlying mechanisms involved in liver fibrosis (Brattin et al., 1985; Lee and Friedman, 2011). By analyzing the liver tissues from model animals with fibrosis, we observed that the molecules related to hypoxia (HIF-1α, HIF-2α) and oxidative stress (Keap1) were increased in hepatocytes, which provided a valid theoretical basis for the *in vitro* research.

Next, to elucidate the underlying mechanisms we chose hepatocytes as the primary target. To simulate the hypoxia *in vitro*, we used 1% oxygen (normoxia with ~20% oxygen) in the incubators similar to Fingas' study (Fingas et al., 2011). In the hepatocytes, we observed that hypoxia only leads to a decrease in PHD1mRNA but not that of its protein expression, which implied that hypoxia controls the expression of PHD1 only at the transcript level. PHD1 is not only sensitive to oxygen concentration but also vulnerable to ROS generation (Kaelin and Ratcliffe, 2008). Our results also showed that knockdown of PHD1 increased the expression of both HIF-2α mRNA and protein in the hepatocytes, but had no effect on HIF-1α level. These findings were consistent with the previous studies reporting HIF-2α as a major downstream mediator of PHD1 during hypoxia tolerance (Aragones et al., 2008; Schneider et al., 2010) and HIF-1α may be regulated by the PHD2 pathway (Takeda et al., 2007). Furthermore, we found that Keap1shRNA could efficiently suppress Keap1 expression and enhance Nrf2 expression, which suggest that Keap1 negatively regulates Nrf2. Therefore, knockdown of Keap1 is vital for promoting Nrf2-mediated cytoprotection.

Until now, most anti-fibrotic approach researches are focused on either alleviating hypoxia or oxidative stress alone, however, a single treatment have showed an unsatisfactory result with a more side-effects (Halliwell, 2013; Kim and Yang, 2015; Biswas, 2016). Considering the interrelation between hypoxia and oxidative stress, in this experiment we used shRNAs to simultaneously knockdown the expressions of both PHD1 and Keap1 so that one sided dominance of either factor was eliminated. Our data suggest that optimal interference of PHD1 or Keap1 was achieved at 12 h in hypoxic condition, therefore the co-transfected cells were cultured in 1% hypoxia up to this time point. We found that loss of PHD1 or Keap1 reduced oxidative stress by activating GSH and by subsequent inhibition of MDA. Schneider et al. reported that knockdown of PHD1 in the hepatocytes led to reduced oxidative stress (Schneider et al., 2010), and inhibition of Keap1 in the hepatocytes improved the resistance against oxidative stress by upregulating Nrf2 (Miyata et al., 2011), which are also inline with our findings and further supported our data. Importantly, our results indicated that combined interference of PHD1 and Keap1 had a synergistic effect on the inhibition of oxidative stress.

Previous studies have shown that apoptosis was involved in the pathogenesis of hepatic fibrosis (Hamdy and El-Demerdash, 2012; Klein et al., 2012). In the study, we observed that apoptosis was decreased in the PHD1-knockdown hypoxic hepatocytes, and the anti-apoptotic effect observed was independent of the HIF signal pathway, which may be associated with the activation of NF-κB (Fitzpatrick et al., 2016). Meanwhile, Hamdy et al. reported that Nrf2 inhibited apoptosis via activation of the anti-apoptotic Bcl-2 protein (Hamdy and El-Demerdash, 2012), and we also demonstrated that knockdown of Keap1 could inhibit the apoptosis of hepatocytes. Furthermore, combined knockdown of PHD1 and Keap1 led to stronger anti-apoptotic effects and improved viability of hepatocytes during hypoxia. Indeed, the hypoxia tolerance in the PHD1-knockdown hepatocytes is mainly as the result of the subsequent decrease in glucose oxidation and oxygen consumption.(Aragones et al., 2008; Schneider et al., 2010).

It is well known that cytokines play central roles in liver fibrosis by regulating inflammatary mediators toward various injuries (Marra, 2002). VEGF contributes to the process of liver fibrosis by promoting angiogenesis and activating HSCs (Yoshiji et al., 2003), TGF-β1 plays a critical role in inducing the epithelial-mesenchymal transition of hepatocytes and thereby promoting collagen synthesis (Gressner and Weiskirchen, 2006; Kaimori et al., 2007), and IGF-1 also stimulates collagen synthesis and HSCs proliferation (Scharf et al., 1998). Ma et al. proposed that both HIF-1α and HIF-2α acted as positive regulators of VEGF (Ma et al., 2014), moreover the changes in TGF-β1 expression was in HIF dependent manner (Qu et al., 2015). However, our data indicated that the levels of the pro-fibrogenic molecules—COL1A1, TGF-β1, VEGF-A, and IGF-1 were downregulated in PHD1 knockdown cells during hypoxia, and this may be because TGF-β1 and VEGF-A may also be under regulation of ROS besides HIF (Kuroki et al., 1996; Jobling et al., 2006). In addition, we found that the knockdown of Keap1 could inhibit the expression of pro-fibrogenic molecules. Furthermore, concomitant knockdown of both PHD1 and Keap1 synergistically reduced the expression of pro-fibrogenic molecules, thereby inhibiting the progression of fibrosis, however, the specific mechanism is still unclear.

Hypoxic hepatocytes are capable of promoting the expression of TGF-β1 and VEGF-A, which further activates HSCs (Qu et al., 2015). In addition, hepatocytes undergoing apoptosis and oxidative stress which in turn activates HSCs (Zhan et al., 2006; Subhadip et al., 2011), Therefore, considering the complex intrahepatic microenvironment and the interaction between hepatocytes and HSCs (Ayako et al., 2004), we used CM from the supernatant of hypoxic hepatocytes to culture HSCs. Our data showed that double-knockdown CM from hepatocytes could suppress the HSCs activation and prevent the progression of fibrosis. Alternatively, prompting apoptosis of activated HSCs may also be an alternative approach to resolve fibrosis (Iredale, 2001). It is worthly noted that double-knockdown CM could induce an increased apoptosis of HSCs, thereby inhibiting their proliferation.

**Figure 8:**
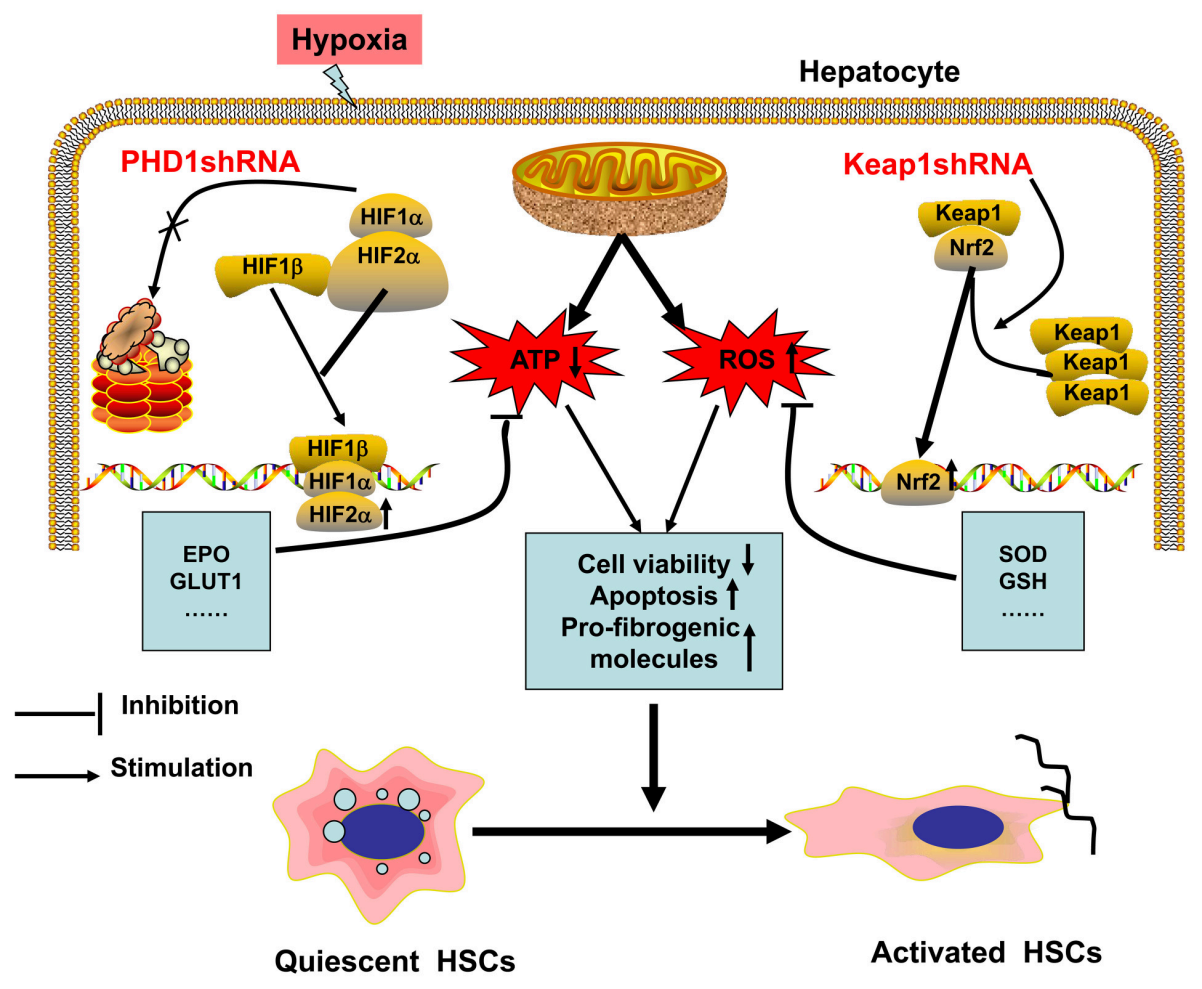
**Regulation model of hepatocytes and HSCs in response to hypoxia**. When hepatocytes are exposed to hypoxia, mitochondrial ATP production decreases while ROS generation increases, which result in HSCs activation. PHD1shRNA and Keap1shRNA mainly facilitate the translocation of HIF-2α and Nrf2 into the nuclei respectively, where the activation of cytoprotective genes reduce hepatocytes injury and weaken the generation of pro-fibrogenic molecules, which finally inhibit the activation of HSCs.

The possible mechanism of hypoxia-induced hepatocytes injury are illustrated in Figure 8. Hypoxia is the initiator of this process, and can induce oxidative stress, which decreases the cellular acitivity, enhances apoptosis, promotes expression of pro-fibrogenic molecules, and hence activates HSC. In our study, we have demonstrated that single-knockdown of PHD1 or Keap1 could alleviate hepatocellular hypoxic injury by upregulating the expression of HIF-2α and Nrf2 respectively, which then inhibits the HSC activation (Figure 8), moreover, double-knockdown of PHD1 and Keap1 could synergistically enhance this effect. Collectively, these findings will provide new targets for developing new effective clinical drugs to treat liver fibrosis. However, further studies are needed to confirm our hypothesis *in vivo*.

## Author contributions

Conceived and designed the experiments: JL, YL, and PC; Performed the experiments: JL, LL, ZW, CS, ZC, XZ, and FD; Analyzed the data and wrote the paper: JL and YL; Revised the paper: YL and PC.

## Funding

This work was financially supported by the National Nature Science Foundation of China (No 81370868), the Fundamental Research Funds for the Central Universities and the Scientific Research Innovation Program for Graduate Students of Jiangsu Province, China (No KYLX16_0298), and (No KYZZ15_0062).

### Conflict of interest statement

The authors declare that the research was conducted in the absence of any commercial or financial relationships that could be construed as a potential conflict of interest.
